# Antipoliovirus Activity of the Organic Extract of *Eupatorium buniifolium*: Isolation of Euparin as an Active Compound

**DOI:** 10.1155/2013/402364

**Published:** 2013-07-17

**Authors:** María Florencia Visintini Jaime, Rodolfo H. Campos, Virginia S. Martino, Lucía V. Cavallaro, Liliana V. Muschietti

**Affiliations:** ^1^Cátedra de Virología, Facultad de Farmacia y Bioquímica, Universidad de Buenos Aires, Junín 956 4 P, 1113 Buenos Aires, Argentina; ^2^Cátedra de Farmacognosia, IQUIMEFA (UBA-CONICET), Facultad de Farmacia y Bioquímica, Universidad de Buenos Aires, Junín 956 2 P, 1113 Buenos Aires, Argentina

## Abstract

The antiviral activity of the organic extract (OE) of *Eupatorium buniifolium* against poliovirus type 1 was determined by *in vitro* assays with an effective concentration 50 (EC_50_) of 23.3 ± 3.3 *µ*g/mL. Bioassay-guided fractionation of the OE allowed the isolation of an active principle that was identified by spectroscopic methods (^1^H- and ^13^C-NMR, EI-MS, UV, and IR spectroscopy) as the benzofuran euparin. The plaque reduction assay in Vero cells was used to assess the antiviral activity of euparin against poliovirus types 1, 2, and 3 with EC_50_ values of 0.47, 0.12, and 0.15 *µ*g/mL, respectively. Moreover, this compound showed high selectivity indexes of 284.9, 1068, and 854.7, respectively. In order to identify the mechanism by which euparin exerts its antiviral activity, the virucidal effect, the pretreatment of Vero cells, and the time of action on one viral replication cycle were evaluated. Results obtained demonstrated that euparin exerts its effect during the early events of the replication cycle, from the virus adsorption to cells up to the first twenty minutes after infection. This is the first report on the presence of euparin in *E. buniifolium* and its antiviral activity.

## 1. Introduction

Poliomyelitis is caused by poliovirus (PV), which can affect the nervous system causing permanent paralysis. PV is an RNA virus that belongs to the genus Enterovirus of the large family of Picornaviridae. There are three serotypes of PV (i.e., PV-1, PV-2, and PV-3). 

Two trivalent polio vaccines have been available since 1961: the intramuscular inactivated vaccine of Salk and the oral attenuated vaccine of Sabin. Both of them stimulate the production of neutralizing antibodies antipolioviruses that protect from the disease. Polioviruses have been eradicated from the United States in 1980 and from the Western Hemisphere in 1991. In 1999, the Global Polio Eradication Initiative wiped out PV-2. Nowadays, and according to the World Health Organization, poliomyelitis remains endemic in Afghanistan, Nigeria, and Pakistan and transmission has been reestablished in three countries which were previously declared as polio-free (Angola, Chad, and the Democratic Republic of the Congo) [1].

In 2006, the “Committee on Development of a Polio Antiviral and Its Potential Role in Global Poliomyelitis Eradication” highlighted the importance of the potential role of an antiviral drug in the context of polio eradication [2] that would be used: (i) for immunodeficient people who are chronically shedding poliovirus, (ii) for people exposed to poliovirus, for example, through unintentional laboratory exposure, (iii) for communities exposed to circulating vaccine-derived poliovirus outbreaks in the posteradication era (likely in conjunction with inactivated polio vaccine).

One strategy for the development of antiviral agents is the search for novel compounds from natural sources. A variety of lead molecules, mainly those isolated from higher plants, have already been reported: terpenoids, flavonoids, coumarins, alkaloids, and lignans [3–6]. Among the numerous medicinal plants growing in our country, *Eupatorium buniifolium* Hook. et Arn. (Asteraceae), popularly known as “romerillo,” “romerillo Colorado,” or “chilca,” is traditionally used as disinfectant and against rheumatic pains [7, 8]. It has been reported to possess *in vitro* anti-inflammatory [9], antioxidant [10], and trypanocidal [11] activities and that some of its extracts can inhibit Herpes simplex virus type I replication [12] and reduce Herpes suis virus viral infectivity [13].

In this study, we report the antiviral activity of the organic extract of *Eupatorium buniifolium *and the isolation and identification of a new antipoliovirus compound, by bioassay-guided fractionation and its possible mechanisms of action. 

## 2. Materials and Methods

### 2.1. Plant Material

The aerial parts of *Eupatorium buniifolium* were collected in Departamento Tafí del Valle in the province of Tucumán, Argentina, in May 2009. The plant material was identified by A. Slanis-B. Juarez and a voucher specimen (Slanis-Juarez 1043 or LIL609703) is deposited at the Herbarium of the Fundación Miguel A. Lillo, University of Tucumán, Argentina.

### 2.2. Extraction of Plant Material

Air-dried and ground aerial parts (500 g) were extracted by maceration with dichloromethane : methanol (1 : 1) for 24 h and then vacuum-filtered. The process was repeated twice and the filtrates were combined and taken to dryness under vacuum to obtain the organic extract (OE). The marc was air-dried and then extracted with water (500 mL) under the same conditions. The resulting aqueous extract (AE) was freeze-dried.

### 2.3. Bioassay-Guided Fractionation of *E. buniifolium* CH_2_Cl_2_ : MeOH (1 : 1) Extract (OE) and Isolation of the Active Compound

The fractionation of OE (30 g) was done by open column chromatography loaded with silicagel 60 (Merck, 0.063–0.2 mm/70–230 mesh; 300 g) and eluted with a step gradient of hexane : ethylacetate (100 : 0 to 0 : 100) and ethylacetate : methanol (100 : 0 to 0 : 100). Ten fractions (250 mL) were obtained and monitored by thin layer chromatography (TLC), carried out on silicagel plates with hexane : ethylacetate (1 : 1) as mobile phase. Fractions with the same chromatographic profile were combined into five fractions (F_1_–F_5_).

Fraction F_2_ (2.1 g) was purified by column chromatography on silicagel 60 (100 g) and eluted with 100% hexane, hexane : ethylacetate (9 : 1; 7 : 3; 1 : 1), 100% ethylacetate, ethylacetate : methanol (1 : 1), and 100% methanol, obtaining 42 fractions of 15 mL each, that were combined afterwards into 17 subfractions (F_2.I_–F_2.XVII_) according to their TLC profile. From fraction F_2.*VI*⁡_, euparin crystallized as yellow needles. Melting point was determined in a Thomas Hoover apparatus. 

### 2.4. Spectrometric Analyses

Euparin was identified by nuclear magnetic resonance ^1^H-NMR and ^13^C-NMR (NMR Varian Inova, 500 MHz in CDCl3), electron impact-mass spectrometry (EI-MS) (Agilent 5973), ultraviolet spectroscopy (UV) (Jasco V-630), and infrared spectroscopy (IR) (Nicolet 380 FT-IR-Smart Multi Bruce HATR, Zn Se 45°).

### 2.5. Cells and Viruses

Vero cells (ATCC CCL 81) were obtained from Asociación Banco Argentino de Células and cultured at 37°C in a 5% CO_2_ incubator in growth medium: Eagle's minimal essential medium (Gibco) supplemented with 10% fetal bovine serum (FBS, PAA), 100 *µ*g/mL streptomycin, 100 IU/mL penicillin, 2 mM L-glutamine, and 100 *µ*M nonessential amino acids (Gibco). Infection Medium (IM), which was used for the antiviral assays, was the same as the growth medium but with 2% FBS. Plaque medium (PM) was IM containing 1% methylcellulose (Sigma). Poliovirus types 1, 2, and 3 (PV-1, PV-2, and PV-3) Sabin strains and herpes simplex virus type 1 (HSV-1) F strain were kindly provided by Dr. María Cecilia Freire (ANLIS-Instituto Dr. Carlos G. Malbrán, Argentina) and Dr. Albert Epstein, respectively. Vesicular stomatitis virus (VSV), Indiana strain (ATCC VR-1421), was purchased from ATCC. All virus stocks were stored at −70°C until used. Viral stocks were propagated and quantified in Vero cells. The number of plaque forming units per mL (PFU/mL) was determined by the plaque assay for virus quantification. 

### 2.6. Viral Cytopathic Effect Reduction Assay

Antiviral activities of *E. buniifolium* extracts (OE and AE) against PV-1 were performed by measuring the reduction of the viral cytopathic effect (CPE). Confluent Vero cells monolayers growing in 96-well plates after 24 h of culture were infected with PV-1 at a multiplicity of infection (m.o.i) of 0.01 PFU/cell in presence of both 25 and 100 *µ*g/mL of OE diluted in dimethyl sulfoxide (DMSO) and AE diluted in sterile distilled water. Control viruses and mock-infected cells were included in each plate. Then, the plate was incubated (37°C, 5% CO_2_) until 90% of viral CPE was reached at control viruses. The reduction of viral CPE was determined by assessment of cell viability by the 3-(4,5-dimethylthiazol-2-yl)-2,5-diphenyltetrazolium bromide (MTS/PMS) assay (CellTiter 96 Aqueous-Promega, Madison, WI) according to the manufacturer's instructions. The absorbance at 490 nm was read in an automatic plate reader (Multi-Mode Microplate Reader-SynergyTM HT-BioTek) according to %  CPE  reduction = [(Abs_treated_ − Abs_control  virus_)/(Abs_cellular  control_ − Abs_control  virus_)]∗100.

### 2.7. Cytotoxicity Assay

Cell viability was assessed by the MTS/PMS test as previously described [14]. Briefly, subconfluent monolayers of Vero cells cultures (8 × 10^3^ cells/well; 24 h) in 96-well plates were exposed to twofold dilutions of OE, fractions F_2_–F_4_, and euparin in DMSO for 72 h at 37°C. Cellular controls were incubated under the same conditions in presence of growth medium without drug. Cell viability was calculated as [Abs_treated  cells_/Abs_cellular  control_] × 100. The cytotoxic concentration 50 (CC_50_) is defined as the concentration that reduces cell viability by 50% with respect to cell control and is determined from dose-response curves. The maximum noncytotoxic concentration (MNCC) is the maximum concentration of OE, fraction, or euparin that leaves 100% of viable cells.

### 2.8. Plaque Reduction Assay

Confluent Vero cell monolayers seeded in 24-well plates (24 h; 5% CO_2_; 37°C) were infected with 100 PFU of PV-1/well in presence of serial twofold dilutions from the MNCC of *E. buniifolium* OE, fractions F_1_–F_5_, or euparin. Following 45 min of adsorption at 37°C, the viral inoculum was removed; cell monolayers were washed twice and overlaid with PM supplemented with the same concentrations of OE, fractions F_1_–F_5_ or euparin added during the adsorption period. Mock-infected cells and virus control were included. After 24–48 h of incubation cell monolayers were fixed and stained with 0.75% crystal violet in methanol : water (40 : 60) and viral plaques were counted. The effective concentration 50 (EC_50_) value is the concentration of OE, fraction, or euparin that reduces the number of viral plaques by 50% with respect to the viral control and was calculated by regression analysis of the dose-response curves generated with the data. Reduction of plaques (%) was calculated as [1 − (no.  plaques_  treated_/no. plaques_control  virus_)] × 100. The selectivity index (SI) was calculated as CC_50_/EC_50_. The antiviral activity of *E. buniifolium* OE and euparin was also determined against PV-2, PV-3, HSV-1, HSV-2, and VSV by reduction viral plaque assays in Vero cells monolayers as described earlier.

### 2.9. Virucidal Assay of Euparin

PV-1 (1.0 × 10^6^ PFU) was incubated at room temperature (r.t.) or 37°C for 30 min in IM with or without 10 *µ*g/mL (10X EC_90_) of euparin. The residual infectious virus particles were determined by viral plaque assays in Vero cell monolayers.

### 2.10. Effect of Vero Cell Pretreatment with Euparin

Pretreatment of Vero cells with euparin was evaluated as previously described [14]. Vero cell monolayers were seeded in 24-well plates. After 24 h (5% CO_2_ incubator, 37°C), cells were washed twice with phosphate buffer saline (PBS) and treated with 1.0 and 10 *µ*g/mL of euparin in IM, corresponding to EC_90_ and 10X EC_90_, respectively. After 7 h incubation at 37°C, the medium was removed; cell monolayers were washed three times with PBS and infected with 100 PFU/well of PV-1 in 0.1 mL of IM. Following 45 min of adsorption at 37°C, the virus inoculum was removed and PM was added. After 24 h, cells were fixed and stained and viral plaques were counted.

### 2.11. Time of Addition Assay of Euparin

To study the effect of euparin in the adsorption and post adsorption events of PV-1 in Vero cells, three different treatments at 1.0 *µ*g/mL (1X EC_90_) were evaluated: (i) only during adsorption period (Adsorption); (ii) after adsorption and until the end of the experiment (After Adsorption), and (iii) during and after the adsorption (Throughout). Briefly, Vero cell monolayers cultured in 24-well plates were precooled for 1 h at 4°C. Cells were then infected with 100 PFU of PV-1 virus/well in presence or absence of euparin and further incubated at 4°C for 1 h. Cell monolayers were washed with PBS, and then PM with or without euparin was added. After 24 h, the viral plaques were counted.

### 2.12. Effect of Time Addition of Euparin on the One-Step Replication Cycle

Confluent Vero cell monolayers cultured in a 24-well plate were infected with PV-1 (m.o.i. = 5) for 1 h at 4°C. Following the adsorption period, cells were washed three times, and 10 *µ*g/mL of euparin was added at different hours after infection (h p.i.): 0, 1, 2, 3, 4, 5, 6, and 7 or at different minutes p.i.: 0, 10, 20, 30, 40, 50, and 60. Cells were further incubated up to 8 h. At this time, the supernatants were collected, clarified by centrifugation (3,500 ×g at 4°C), and the extracellular virus production was determined by viral plaque assays. 

### 2.13. Statistical Analysis

Data are presented as means ± SD of three independent assays. The EC_50_ and CC_50_ values were calculated using GraphPad Prism programme v. 5.01. Statistical differences between different treatments were determined using one way ANOVA with Tukey's posttest. Differences were considered significant when the *P* value was less than 0.05. 

## 3. Results

### 3.1. Antiviral Activity of *E. buniifolium* OE and AE against PV-1

The antiviral activity of *E. buniifolium* OE and AE was determined against PV-1 by the viral cytopathic effect reduction assay. The results obtained showed that only OE inhibited PV-1 replication at the two concentrations tested (25 and 100 *µ*g/mL) with CPE reduction values higher than 50% (data not shown).

### 3.2. Bioassay Guided Fractionation of *E. buniifolium* OE and Structure Elucidation of Euparin

**Figure 1:**
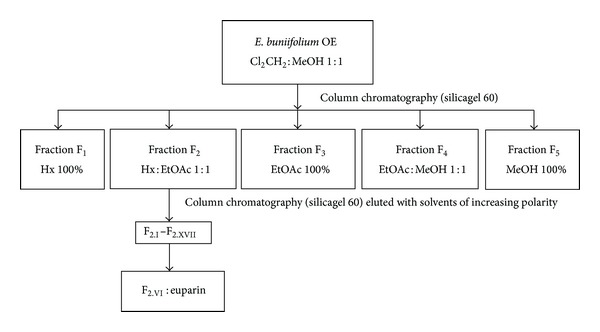
Bioassay-guided fractionation of *Eupatorium buniifolium* organic extract (OE).

**Figure 2:**
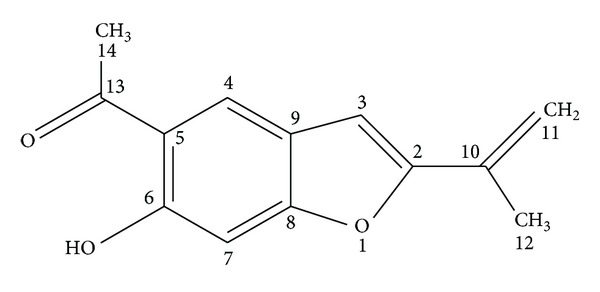
Chemical structure of euparin: 1-[6-hydroxy-2-(1-methylethenyl)-5-benzofuranyl] ethanone, C_13_H_12_O_3_, MW: 216.23.

Fractionation of OE, by chromatographic techniques, yielded five fractions (F_1_–F_5_) which were assayed for their *in vitro* anti-PV-1 activity by the plaque reduction assay (Figure 1 and Table 1). Further purification of F_2_ yielded subfractions F_2.I_–F_2.XVII_ (Figure 1). From fraction F_2. *VI*⁡_ a major anti-PV compound (39 mg; 130.0 mg/100 g dried extract) was isolated and identified as euparin (purity > 95%, by High Performance Liquid Chromatography (HPLC); m.p. 120–122°C) by comparison of its spectral data with the literature values (Figure 2) [15, 16].

### 3.3. Spectral Data of Euparin

Euparin presented the following spectral data: UV *λ*_max_ (nm) MeOH: 260, 356; IR (KBr), *ν*_max⁡_ (cm^−1^): 3388 (OH), 1470 (aromatic), 1636 (C=COR); ^1^H NMR (MeOH-*d*4), *δ* (ppm): 8.18 (1H, s, H-4); 6.95 (1H, d, *J* = 0.5 Hz H3); 6.78 (1H, s, H7); 5.74 (1H, m, H11a); 5.21 (1H, m, H11b); 2.72 (3H, s, H-14); 2.14 (3H, s, H-12). ^13^C NMR (DMSO-*d6*), *δ* (ppm): 204.60 (s, C-13), 161.24 (s, C-8), 159.49 (s, C-6), 157.64 (s, C-2), 132.45 (s, C-5), 124.27 (d, C-4), 121.91 (s, C-9), 116.79 (s, C-10), 112.23 (t, C-11), 102.54 (d, C-3), 98.24 (d, C-7), 25.59 (q, C-14), 17.85 (q, C-12); EI-MS (*m*/*z*, rel.int.) 218 (19.30), 217 (48.18), 216 (68.16) [M]^+^, 203 (2.52), 202 (16.22), 201 (100.00), 173 (26.88). 

### 3.4. Cytotoxicity Assay

The cytotoxic effect of *E. bunnifolium* OE, fraction F_2_ and euparin, on Vero cells, was evaluated by the MTS/PMS assay and expressed as cell viability percentage. When cells were treated with OE, fraction F_2_, or euparin, CC_50_ values were 114.8 ±1.8 *µ*g/mL, 254.2 ± 1.2 *µ*g/mL, and 128.2 ± 2.2 *µ*g/mL, respectively (Table 1).

### 3.5. Antiviral Activity

The antiviral activity of *E. buniifolium* organic extract (OE), fractions F_1_–F_5_, and euparin against PV-1 was evaluated by the plaque reduction assay. As showed in Table 1, EC_50_ values for OE, fraction F_2_, and euparin were 23.3 ± 3.3 *μ*g/mL; 13.3 ± 2.6 *μ*g/mL and 0.47 ± 0.05 *μ*g/mL, respectively. The SI values for OE, F_2_, and euparin were 5.5, 19 and 284.9, respectively. 

The antiviral activity of OE and euparin was also evaluated against PV-2, PV-3, HSV-1, HSV-2, and VSV by the plaque reduction assays. Results showed that OE was active against PV-2, and PV-3 with EC_50_ values of 25.0 ± 1.8 *μ*g/mL and 21.1 ± 2.5 *μ*g/mL, respectively. Euparin showed EC_50_ values of 0.12 ± 0.01 *µ*g/mL (SI = 1068) and 0.15 ± 0.01 *µ*g/mL (SI = 854.7) against PV-2 and PV-3 respectively (Table 1). Neither *E. buniifolium* OE nor euparin showed antiviral activity against HSV-1, HSV-2, and VSV.

### 3.6. Virucidal Activity and Effect of Vero Cells Pretreatment with Euparin on PV-1 Infection

**Figure 3:**
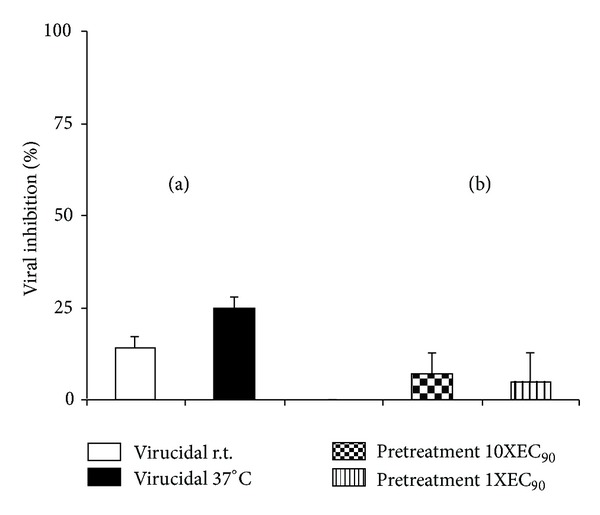
Virucidal activity (a) and the effect of pretreatment of Vero cells with euparin (b). The virucidal activity of 10 *µ*g/mL (10X EC_90_) at 37°C and r.t. and the pretreatment with 1.0 and 10 *µ*g/mL were evaluated against PV-1. Data represent % of virus inhibition compared to untreated controls as mean ± SD (*n* = 3). Experiments were done in quadruplicate.

No significant reduction of viral infectivity was observed when the viruses were in direct contact (treated) with concentrations of euparin as high as 10 *µ*g/mL (10X EC_90_). These results suggested that euparin did not exert a virucidal activity against PV-1 (Figure 3(a)).

Moreover, the addition of 1.0 and 10 *µ*g/mL of euparin to Vero cells monolayers 7 h before infection with PV-1 did not exert any reduction in viral infection. Thus, it can be deduced that no antiviral activity was induced in these cells during the pretreatment with euparin (Figure 3(b)).

### 3.7. Time of Addition of Euparin

**Figure 4:**
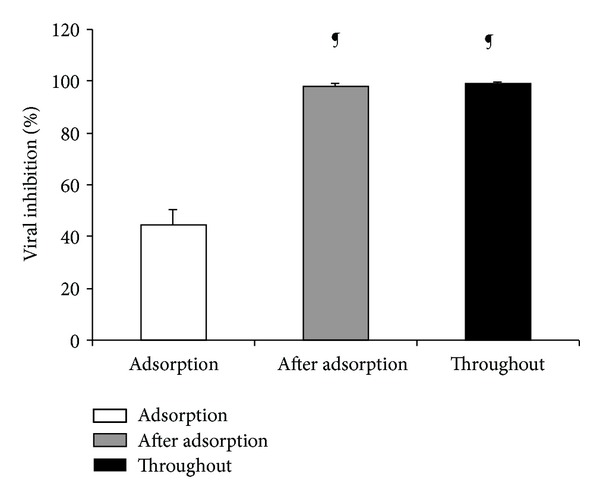
Percentage of viral inhibition against PV-1 under different experimental conditions. Euparin (1.0 *µ*g/mL) was present at the indicated times. Data represents % of virus inhibition obtained at each condition with respect to untreated control as mean ± SD (*n* = 3). Experiments were done in quadruplicate. ^¶^ *P* < 0.001 versus Adsorption.

To determine the time of the viral cycle at which euparin interferes with viral replication, an assay was carried out by adding 1.0 *µ*g/mL (1X EC_90_) of this compound during the different infection periods. The maximum inhibition was obtained when the compound was present in the postadsorption period or throughout the infection time (Figure 4). 

### 3.8. Effect of Time Addition of Euparin on the One-Step Viral Replication Cycle of PV-1

**Figure 5:**
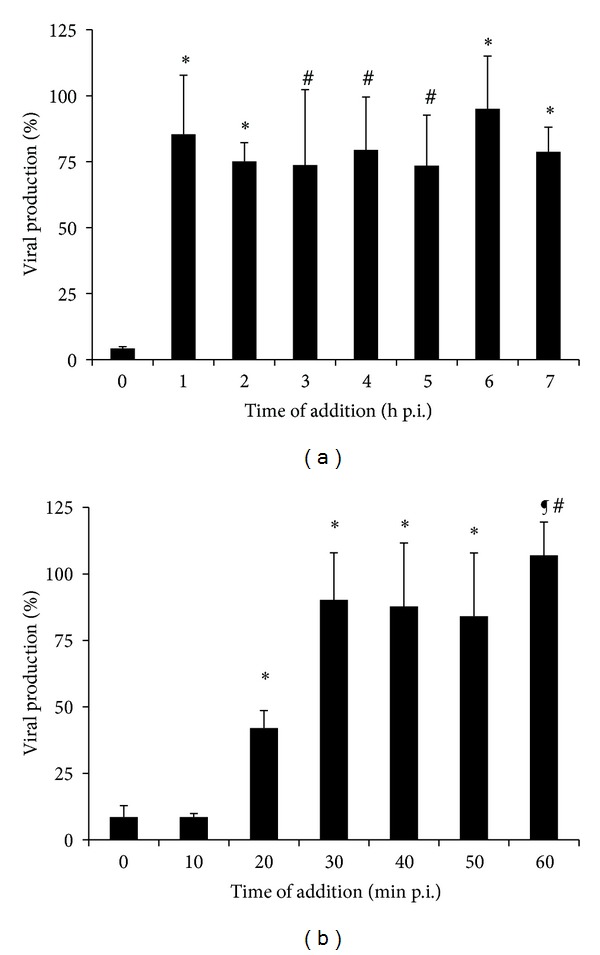
Effect of addition of euparin on the PV-1 production during a one-step replication cycle. Vero cell monolayers were infected at m.o.i. = 5 and euparin (10 *µ*g/mL) was added at different h p.i. (a) or min p.i. (b), after the adsorption period. At 8 h p.i. at 37°C, the extracellular PV-1 production was determined by the viral plaque assay. *P < 0.01 versus 0 h p.i.; versus 0 min p.i.; versus 10 min p.i. ^#^ *P* < 0.05 versus 0 h p.i.; versus 0 min p.i. ^¶^ *P* < 0.001 versus 0 min p.i.; 10 min p.i.

The effect of addition of euparin on one cycle of virus replication was evaluated to define which step of PV-1 replication is inhibited. Euparin was added at different h p.i. and then extracellular viral production was evaluated at 8 h p.i. Viral titers were markedly reduced when the compound was added during the first hour p.i., after which this effect was not observed, suggesting that euparin may be acting at a time point between 0 and 1 h p.i. (Figure 5(a)). The same experiment was repeated but adding it during the first h p.i., every 10 min. The results (Figure 5(b)) showed that euparin is needed to be present before the first 20 min p.i. to obtain the maximal inhibitory effect.

## 4. Discussion

Throughout history, medicinal plants have been widely used to treat a great variety of diseases, and the majority of new drugs have been developed from natural products and from compounds derived from these sources. The research and development of new natural compounds with antiviral activity have attracted the researchers' interest [17, 18] as good candidates suitable for further modification and optimization of bioactivity.

In this context, we have evaluated the antiviral activity of the medicinal plant *E. buniifolium* against PV-1. Among the extracts tested, only the organic extract (OE) exerted antiviral activity (EC_50_ = 23.3 ± 3.3 *μ*g/mL). 

This finding prompted us to perform a bioassay-guided fractionation of OE by chromatographic techniques. Among all the fractions obtained (F_1_–F_5_), F_2_ showed the highest antiviral activity (EC_50_ = 13.3 ± 2.6 *μ*g/mL) and was selected for further purification. From this fraction a bioactive compound was isolated and identified as the benzofuran euparin (EC_50_ = 0.47 ± 0.05 *μ*g/mL). The EC_50_ values obtained for OE, fraction F_2_, and euparin demonstrated that a progressive increase in the anti PV-1 activity was gained during the purification process.

Euparin was first isolated from *Eupatorium purpureum* [19] and its chemical structure was defined as a benzofuran by Kamthong and Robertson in 1939 [20].

Natural benzofurans are heterocyclic compounds frequently synthesized by species of the Asteraceae family. Benzofurans and their derivatives show a wide range of pharmacological properties such as antiviral, antimicrobial, antifungal, anti-inflammatory, antiallergic, and cytotoxic activities [21–27]. Although many biological assays employing euparin have been undertaken, only a few pharmacological activities have been demonstrated [28, 29]. However, its antiviral activity had not been evaluated so far.

Euparin showed low cytotoxicity on Vero cells while exerting a significant antiviral activity against the three types of polioviruses (PV-1, PV-2, and PV-3), and consequently high selectivity has been demonstrated with SI values of 284.9, 1068, and 854.7, respectively. 

These results prompted us to characterize the probable mechanism of action. As euparin showed neither virucidal activity nor protection of the cells in the pretreatment, we decided to evaluate which step of viral cycle was inhibited by this compound.

Time of addition assays demonstrated that euparin completely inhibited the viral plaques formation when it was added in the postadsorption period. This result was later confirmed with time of addition assay on one-step replication viral cycle where we observed that the maximum inhibition was obtained when euparin was added during the first twenty minutes after infection. This inhibition was abolished when euparin was added beyond that time. These results suggest that the likely euparin mechanism of action would be the interference with one of the first events of poliovirus replication cycle probably on the penetration and/or uncoating process and not the viral RNA replication step. We could speculate that euparin exert a similar mechanism of action as other antiviral compounds called capsid binders as the WIN compounds [30]. Euparin, as well as these compounds (i.e., V-073), could bind at the innermost end of the hydrophobic pocket within VP1 [24], but it does not prevent attachment of the virus to the host cell, leading to a more rigid capsid making the virus more resistant to uncoating, thus preventing the RNA delivery to the cellular cytoplasm. Further studies are necessary to properly define the mechanism of action of euparin.

In addition, the high selectivity activity against poliovirus in the submicromolar order, specially PV-1, and the specificity of euparin among the evaluated viruses make this an interesting compound for the development of new antipoliovirus drugs since many prototype drugs under clinical trials, as pleconaril, are unable to inhibit PV-1 [31].

## 5. Conclusions

In this study we report the antipoliovirus activity of *Eupatorium buniifolium* and the isolation of euparin, as the active principle responsible for this activity. The results obtained contribute to describe a new biological activity in a known compound and suggest that euparin emerges as a new selective antipoliovirus drug or as a lead molecule for the developing of more potent derivatives. To our knowledge, this is the first report describing the presence of euparin in *E. buniifolium* and also the first detection of its antiviral activity. Further studies are being undertaken to define the molecular target of euparin.

## Supplementary Material

1H-NMR (MeOH-d4) and 13C-NMR (DMSO-d6) spectra of euparin.

## Figures and Tables

**Table 1:** Cytotoxicity and antiviral activity of extracts, fractions, and euparin against PV-1, PV-2, and PV-3.

	CC_50_ (*µ*g/mL)	PV-1			PV-2			PV-3		
		EC_50_ (*μ*g/mL)	EC_90_ (*μ*g/mL)	SI	EC_50_ (*μ*g/mL)	EC_90_ (*μ*g/mL)	SI	EC_50_ (*μ*g/mL)	EC_90_ (*μ*g/mL)	SI
*E. buniifolium* OE	114.8 ± 1.8	23.3 ± 3.3	47.2 ± 2.8	5.5	25.0 ± 1.8	41.5 ± 3.4	4.6	21.1 ± 2.5	38.8 ± 5.7	4.8
*E. buniifolium* AE	>2000	Inactive	—	—	Inactive	—	—	Inactive	—	—
Fraction F_1_	n.d.	Inactive	—	—	n.d.	—	—	n.d.	—	—
Fraction F_2_	254.2 ± 1.2	13.3 ± 2.6	32.6 ± 1.4	19	n.d.	—	—	n.d.	—	—
Fraction F_3_	140.8 ± 2.7	27.8 ± 1.1	43.3 ± 2.4	5.1	n.d.	—	—	n.d.	—	—
Fraction F_4_	205.1 ± 3.4	19.4 ± 1.6	27.2 ± 0.2	10.6	n.d.	—	—	n.d.	—	—
Fraction F_5_	n.d.	Inactive	—	—	n.d.	—	—	n.d.	—	—
Euparin	128.2 ± 2.2	0.47 ± 0.05	1.01 ± 0.01	284.9	0.12 ± 0.01	0.52 ± 0.01	1068	0.15 ± 0.01	0.36 ± 0.07	854.7

The maximal concentration tested for antiviral activity was 100 *μ*g/mL; the extract or fractions were considered inactive if they were unable to inhibit viral replication at this concentration.

n.d.: not determined.

CC_50_: concentration that reduces cell viability by 50% with respect to cell control; EC_50_: concentration that reduces the number of viral plaques by 50% with respect to the viral control; EC_90_: concentration that reduces the number of viral plaques by 90% with respect to the viral control; SI = CC_50_/EC_50_.
