# Supplementary material for: Antipoliovirus Activity of the Organic Extract of Eupatorium buniifolium: Isolation of Euparin as an Active Compound
Source: Evid Based Complement Alternat Med. 2013 Jul 17;2013:402364. doi: 10.1155/2013/402364 (PMC3730360; doi:10.1155/2013/402364)
Supplement: Supplementary file 1 — 1H-NMR (MeOH-d4) and 13C-NMR (DMSO-d6) spectra of euparin. [file 402364.f1.pdf]

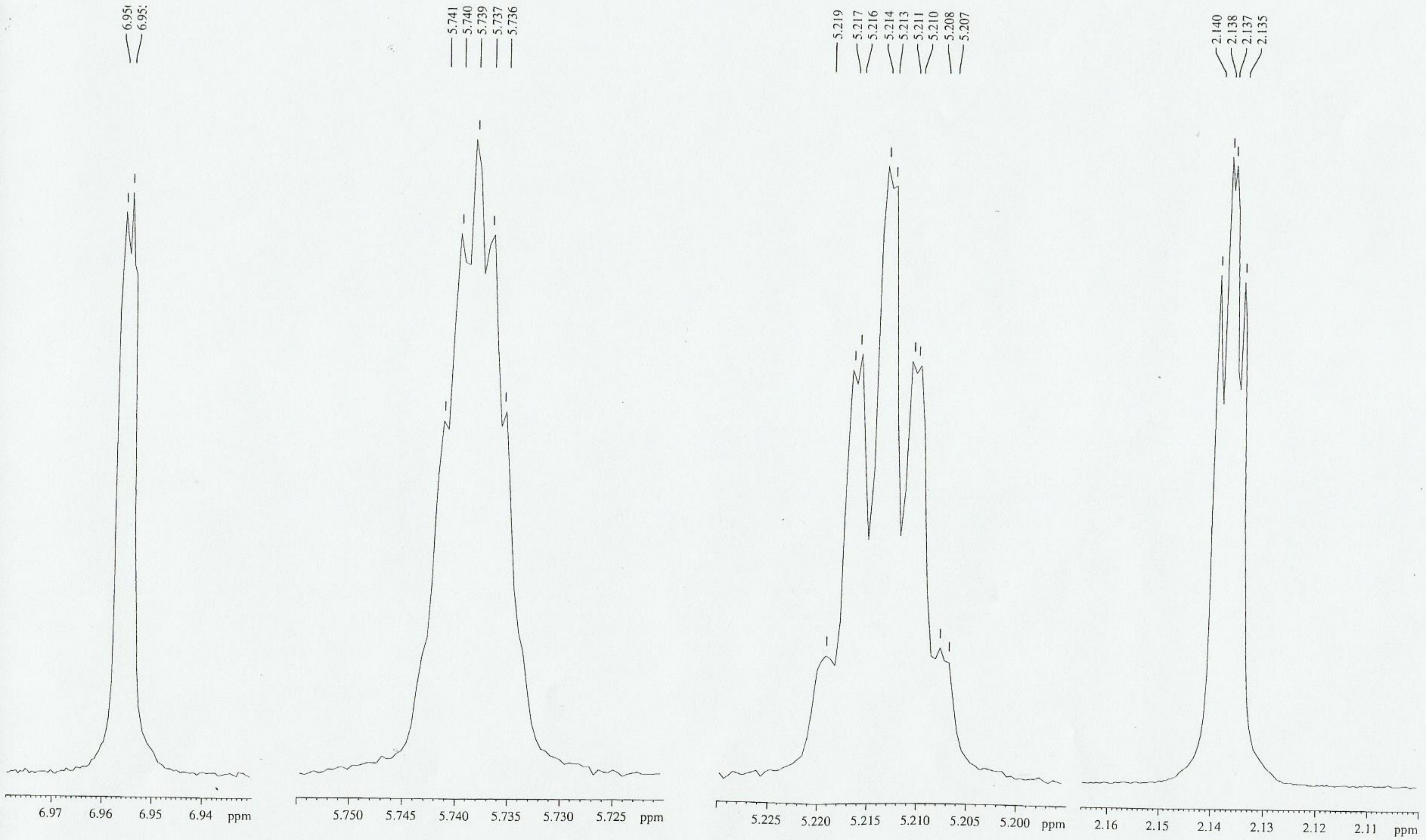

BRUKER

Flavo EB

Current Data Parameters
NAME U11–041206
EXPNO 1
PROCNO 1

F2 – Acquisition Parameters
Date_ 2011–05–14
Time 18.28
INSTRUM Avance 500
PROBHD 5 mm PABBI 1H/
PULPROG zg30
TD 65536
SOLVENT MeOD
NS 16
DS 2
SWH 7500.000 Hz
FIDRES 0.114441 Hz
AQ 4.3691168 sec
RG 161
DW 66.667 usec
DE 6.00 usec
TE 298.1 K
D1 1.00000000 sec
TD0 1

===== CHANNEL f1 =====
NUC1 1H
P1 7.00 usec
PL1 0 dB
PL1W 37.67829514 W
SFO1 500.1327507 MHz

F2 – Processing parameters
SI 32768
SF 500.1300000 MHz
WDW EM
SSB 0
LB 0.10 Hz
GB 0
PC 1.00
