# Supplementary material for: Antipoliovirus Activity of the Organic Extract of Eupatorium buniifolium: Isolation of Euparin as an Active Compound
Source: Evid Based Complement Alternat Med. 2013 Jul 17;2013:402364. doi: 10.1155/2013/402364 (PMC3730360; doi:10.1155/2013/402364)
Supplement: Supplementary file 3 [file 402364.f3.pdf]

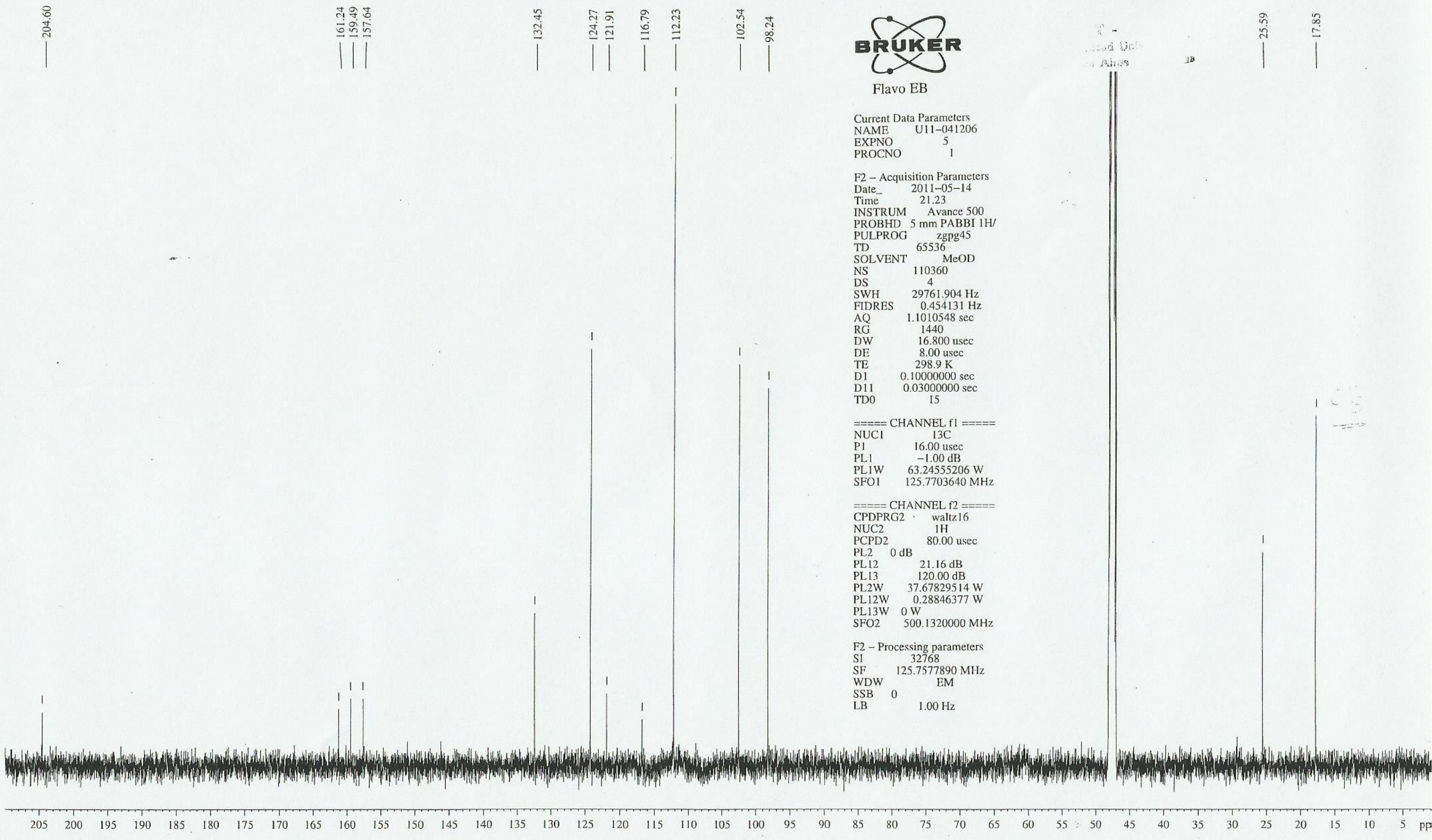
204.60
161.24
159.49
157.64
132.45
124.27
121.91
116.79
112.23
102.54
98.24
25.59
17.85
BRUKER
Flavo EB
Current Data Parameters
NAME U11-041206
EXPNO 5
PROCNO 1
F2 - Acquisition Parameters
Date_ 2011-05-14
Time 21.23
INSTRUM Avance 500
PROBHD 5 mm PABBI 1H/
PULPROG zgpg45
TD 65536
SOLVENT MeOD
NS 110360
DS 4
SWH 29761.904 Hz
FIDRES 0.454131 Hz
AQ 1.1010548 sec
RG 1440
DW 16.800 usec
DE 8.00 usec
TE 298.9 K
D1 0.10000000 sec
D11 0.03000000 sec
TD0 15
===== CHANNEL f1 =====
NUC1 13C
P1 16.00 usec
PL1 -1.00 dB
PL1W 63.24555206 W
SFO1 125.7703640 MHz
===== CHANNEL f2 =====
CPDPRG2 waltz16
NUC2 1H
PCPD2 80.00 usec
PL2 0 dB
PL12 21.16 dB
PL13 120.00 dB
PL2W 37.67829514 W
PL12W 0.28846377 W
PL13W 0 W
SFO2 500.1320000 MHz
F2 - Processing parameters
SI 32768
SF 125.7577890 MHz
WDW EM
SSB 0
LB 1.00 Hz
205 200 195 190 185 180 175 170 165 160 155 150 145 140 135 130 125 120 115 110 105 100 95 90 85 80 75 70 65 60 55 50 45 40 35 30 25 20 15 10 5 ppm
